# Pediatric living donor liver transplantation (LDLT): Short- and long-term outcomes during sixteen years period at a single centre- A retrospective cohort study

**DOI:** 10.1016/j.amsu.2022.103938

**Published:** 2022-06-07

**Authors:** Emad Hamdy Gad, Ahmed Nabil Sallam, Hosam Soliman, Tarek Ibrahim, Tahany Abdel Hameed Salem, Mohammed Abdel-Hafez Ali, Mohammed Al-Sayed Abd-same, Islam Ayoub

**Affiliations:** a Hepatobiliary Surgery, National Liver Institute, Menoufia University, Shebeen Elkoum, Egypt; b Pediatric Hepatology, National Liver Institute, Menoufia University, Shebeen Elkoum, Egypt; c Radiology, National Liver Institute, Menoufiya University, Shebeen Elkoum, Egypt

**Keywords:** LDLT, Pediatric LT, Morbidity, Mortality

## Abstract

**Background and objectives:**

Pediatric living donor liver transplantation (LDLT) is an effective tool for managing pediatric patients with end-stage liver disease (ESLD) with good long-term graft and patient survival, especially after improvement in peri-operative care, surgical tools and techniques; however, the morbidity and mortality after such a procedure are still a challenging matter. The study aimed to analyze short-and long-term outcomes after pediatric LDLT in a single centre.

**Methods:**

We retrospectively analyzed 67 pediatric patients who underwent LDLT in the period from April 2003 to July 2018. The overall male/female ratio was 40/27.

**Results:**

Forty-one (61.2%) of patients had ≥1 early and/or late morbidities; the early (less than 3months) and late (≥3months) ones affected 36(53.7%) and 12(17.9%) of them respectively. The 16-year graft and patient survivals were 35(52.2%) while early and late mortalities were 23(34.3%) and 9(13.4%) respectively. Sepsis and chronic rejection were the most frequent causes of early and late mortalities respectively. Moreover, more packed RBCs transfusion units, bacterial infections, and pulmonary complications were independent predictors of poor patient survival.

**Conclusions:**

More packed RBCs transfusion units intra-operatively, and post-liver transplant (LT) bacterial infection, sepsis, chronic rejection, as well as pulmonary complications had a negative insult on our patients' outcomes, so proper management of them is mandatory for improving outcomes after pediatric LDLT.

## Highlights

• More packed RBCs transfusion units intra-operatively had a negative insult on outcomes after paediatric LDLT.
• Bacterial infection and pulmonary complications led to poor outcomes after paediatric LDLT.
• Sepsis and chronic rejection led to post paediatric LDLT poor patient outcomes.

## List of abbreviations

ACS: Abdominal compartment syndrome
AIH: Autoimmune hepatitis
APTT: Activated partial thromboplastin time
ARDS: Acute respiratory distress syndrome
BA: Biliary atresia
BCS: Budd Chiari syndrome
BMI: Body mass index
CIT: Cold ischemia time
CT: Computed tomography
CTP: Child-Turcotte-Pugh
CUSA: Cavitron ultrasonic surgical aspirator
DDLT: Deceased donor liver transplantation
D-D: Duct to duct
ERCP: Endoscopic retrograde cholangeopancreatography
ESLD: End-stage liver disease
GIT: Gastrointestinal
GRWR: Graft recipient weight ratio
GV/SLV: Graft volume to the recipient's standard liver volume
HA: Hepatic artery
HAT: Hepatic artery thrombosis
HCV: Hepatitis C virus
HJ: Hepaticojejunostomy
HV: Hepatic veins
HVT: Hepatic vein thrombosis
IOC: Intra-operative cholangiography
IVC: Inferior vena cava
LDLT: Living donor liver transplantation
LFSG: Large for size graft
LFT: Liver function tests
LL: Left lobe
LT: Liver transplantation
MELD: Model for end-stage liver disease
MHV: Middle hepatic vein
MMF: Mycophenolate mofetil
MRCP: Magnetic resonance cholangeopancreatography
OV: Oesophagal varices
PELD: Pediatric end-stage liver disease
PHG: Portal hypertensive gastropathy
PHN: Portal hypertension
POD: Postoperative day
PTD: Percutaneous transhepatic drainage
PV: Portal veins
PVT: Portal vein thrombosis
RBCs: Red blood cells
RL: Right lobe
SFSG: Small for size graft
US: Ultrasound
WIT: Warm ischemia time

## 1 Introduction

Living donor liver transplantation (LDLT) has become the gold standard treatment option for paediatrics with end-stage liver disease (ESLD), especially after improved patient selection, increased experience, advancement in (pediatric anaesthesia, surgical techniques, graft preservation, peri-operative and intensive care, medical management, antimicrobial medications as well as immunosuppressive agents) [[1], [2], [3], [4]]. However, the complication rate after such a pediatric procedure is still high with a negative insult on transplanted grafts, pediatric recipient morbidities and mortalities [[5], [6], [7]]**.**

Those complications can be categorized into short-term (early; less than 3months) and long-term (late; ≥ 3months) ones [4,[8], [9], [10], [11], [12]]**.** Moreover, they include post-transplant pulmonary, vascular, biliary, neurological, and infectious complications, as well as acute rejection, chronic rejection, renal dysfunction, etc [1,[12], [13], [14], [15], [16], [17], [18], [19], [20], [21]]**.**

They should be prevented, and if occurred; should be diagnosed and managed early to improve graft and patient outcomes, however, those outcomes are affected also by additional variables (i.e. Large for size graft (LFSG), pediatric end-stage liver disease (PELD)/model for end-stage liver disease (MELD) scores, centre experience/volume, operative time, operative blood loss, blood transfusion units, etc); those variables should be modulated also for getting better short-and long-term outcomes [8,[22], [23], [24], [25], [26]]**.**

To our knowledge; the short- and long-term outcomes after pediatric LDLT is few in literature studies, so we analyzed this issue in a single tertiary Egyptian centre for 16 years period.

## 2 Pediatric recipients and methods

We did this cohort study that analyzed short- and long-term outcomes after pediatric LDLT after being approved by our institutional review board and after obtaining written informed consent regarding surgeries and research from both the recipients' parents/Guardians and the donors. It was performed in the department of hepato-pancreato-biliary surgery, National liver institute, University of Menoufiya, Menoufiya, Egypt during the period from April 2003 to June 2019(the liver transplantation (LT) operations were done between April 2003 and July 2018 and the follow-up started from POD1 until June 2019 or until patient loss(median: 18 months; range(0.03–194 months))).

Our series involved 67 pediatric recipients (less than 18years) after exclusion of adults, recipients with data loss, and cases who refused research. Our work was registered in the research registry with registration NO of researchregistry4593 (www.researchregistry.com) and it was reported in line with the STROCSS criteria [27].

**Fig. 1:**
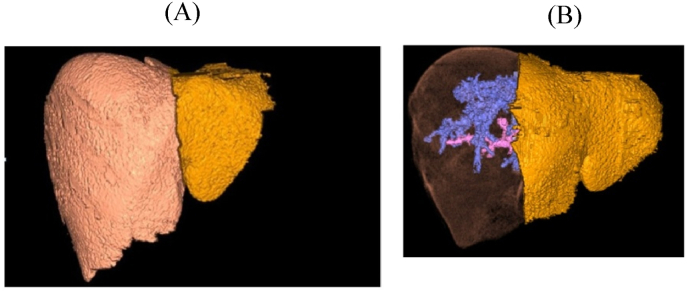
CT with hepatic protocol and 3d imaging reconstruction showing: A: Left lateral graft(yellow) with expected GRWR of 2.2(chosen). B: Left lobe graft(yellow) with expected GRWR of 4.9(Excluded to avoid LFSG). (For interpretation of the references to colour in this figure legend, the reader is referred to the Web version of this article.)

**Fig. 2:**
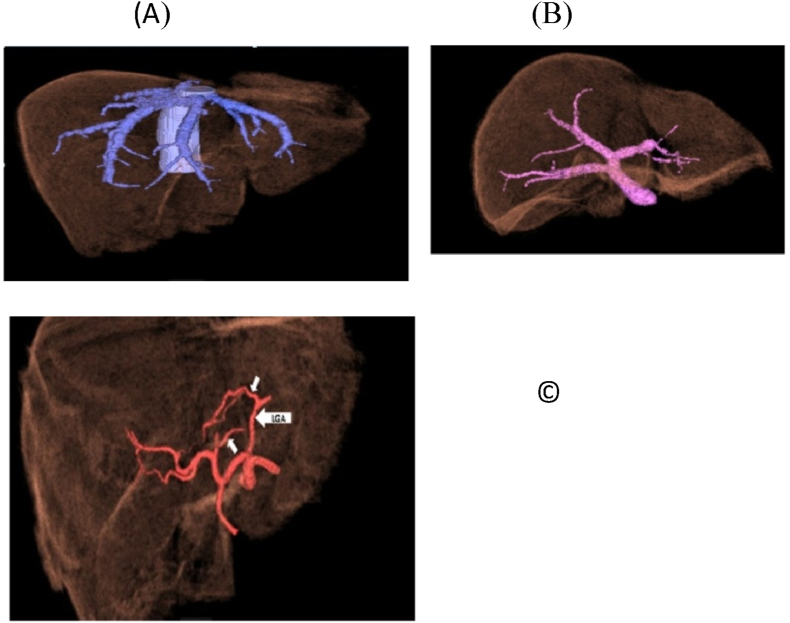
CT with hepatic protocol and 3d imaging reconstruction showing: A: separate left HV. B: single left PV. C: 2 left HAs.

All donors were ≥19.5 years old and their assessment included clinical assessment, psychological assessment, lab studies (liver function tests (LFT), virology, etc), abdominal ultrasound (US), computed tomography (CT angiography and CT volumetric studies), magnetic resonance cholangeopancreatography(MRCP), liver biopsy, etc. In late cases; we did CT with hepatic protocol and 3d imaging reconstruction for determining liver graft volume and vascular variations, moreover; we did our best to avoid cases with an estimated graft recipient weight ratio (GRWR) less than 0.8, as well as GRWR>4 to avoid small for size graft (SFSG), and LFSG respectively for being away from their bad sequels Figure (1: A, B), Figure (2: A, B, C).

The study parameters were collected from a prospectively maintained database in our LT unit and were analyzed retrospectively. Those parameters included pediatric recipients' pre-and intra-operative variables, their donors' variables, primary liver diseases, and postoperative measures.

The details of donors' and recipients' surgical techniques including recipients' vascular and biliary reconstructions have been described previously [[28], [29], [30]]**.** In short; in the donor surgery; the graft type was chosen concerning the estimated GRWR, and the ratio of the graft volume to the recipient's standard liver volume (GV/SLV), furthermore, the hepatectomy was done using Cavitron ultrasonic surgical aspirator (CUSA) device. The donor biliary anatomy was determined according to both the pre-operative MRCP, and the intra-operative cholangiography(IOC), while the vascular anatomy depended upon pre-operative CT angiography ± intra-operative Doppler US. On the other hand, in the recipient surgery, the total hepatectomy phase was done with meticulous dissection and good hemostasis especially in cases with PHN to decrease blood loss, moreover; the hilar portal structures dissection was performed near the liver for obtaining the maximum length of those structures for better future reconstruction, also, the inferior vena cava (IVC) was carefully preserved with temporary portocaval shunts in some cases.

**Fig. 3:**
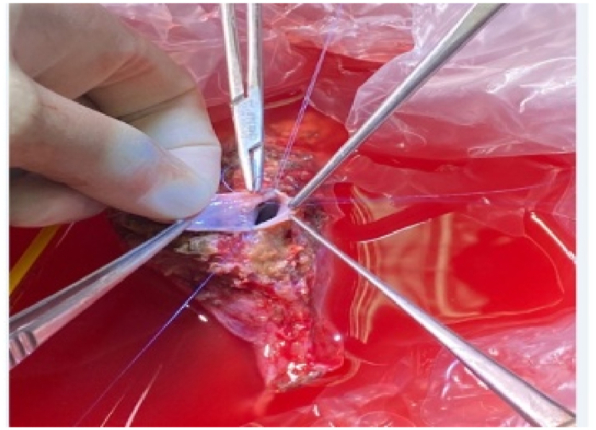
Performing HV patch graft on the back table.

**Fig. 4:**
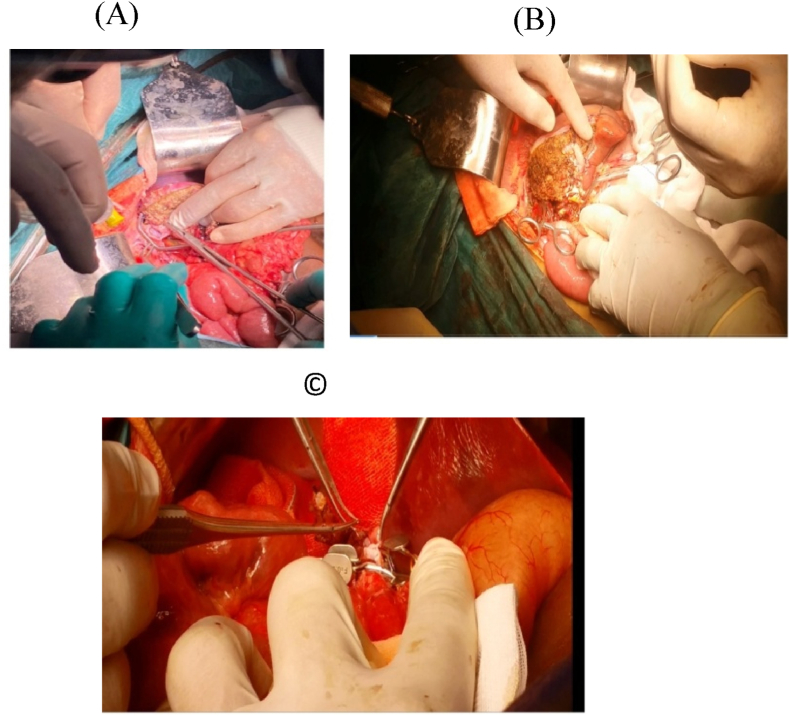
Implantation phase: A: HV reconstruction, B: PV reconstruction, C: HA reconstruction.

**Fig. 5:**
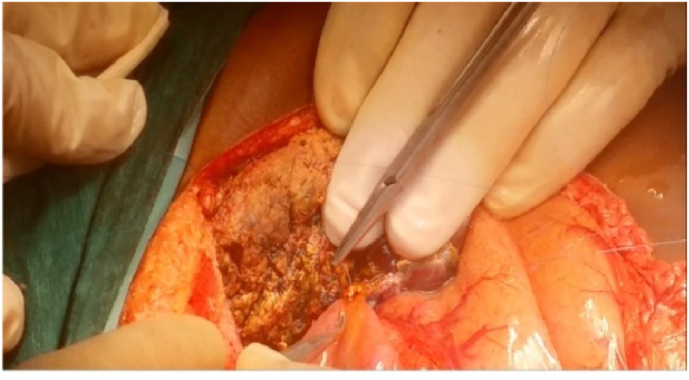
Implantation phase: HJ reconstruction.

On the other hand; on the back table, Hydroxyl tryptophan ketoglutarate solution was used for graft preservation with vascular manipulations of its hepatic veins(HV)/portal veins(PV) in some cases; Fig. 3. Then in the implantation phase; HV and PV anastomoses were performed with the aid of surgical loupes using continuous 5/0 and 6/0 prolene sutures respectively; Fig. 4: A, B; moreover, PV anastomosis was done with a growth factor. Then, the hepatic artery (HA) anastomosis was achieved with the help of surgical loupes or microscopy using interrupted 8/0 prolene stitches; Fig. 4:C. The biliary anastomoses were done with the aid of surgical loupes using interrupted 6–0 prolene/Polydioxanone(PDS) stitches; Fig. 5. Doppler US was done routinely after vascular reconstruction and after abdominal closure to determine the pattern and velocity of blood flow. Finally, all our recipients' abdomens were closed primarily without the occurrence of any abdominal compartment syndrome (ACS).

The post-operative measures have been described previously [[28], [29], [30]]**.** In brief; they included: 1- Immunosuppression therapy and protocol; it consisted of tacrolimus(FK506) and prednisolone, however, some cases were given cyclosporine when side effects (i.e. neurotoxicity or nephrotoxicity) developed with tacrolimus. Mycophenolate mofetil(MMF) was given for multiple episodes of acute rejection, chronic rejection, and for decreasing tacrolimus dose to prevent or treat renal impairment. On the other hand, sirolimus and/or everolimus were given to some patients to replace tacrolimus if side effects developed and to treat chronic rejection. Lastly, an interleukin-2 receptor blocker was given in late cases at POD 0 and 4 for minimizing the tacrolimus dose.

2- To prevent infection; antibacterial (pre-operative 3rd generation cephalosporine, then intra-/post-operative Imepanem + metronidazole until culture result), antifungal (fluconazole), and antiviral (acyclovir) were given. 3- For prophylaxis of vascular thromboses; Heparin infusion was given (dose; 180–200units/kg/day) adjusted according to activated partial thromboplastin time (APTT) (target levels; 50–70 s), then acetylsalicylate and dipyridamole were given at POD8 at doses of 2 mg/kg/d and 4 mg/kg/d respectively for 3 months.

4-The follow-up of pediatric recipients (i.e. by transplant surgeons, pediatric hepatologists, pediatric endoscopists, and pediatric intervention radiologists) was done daily until hospital discharge, then weekly until the end of the 1st 3 months then monthly until the end of the 1st year, then yearly until the end of the follow-up period to detect: a-Early (short-term; less than 3months), and late (Long-term; ≥3months) morbidities(i.e. infection, pulmonary, vascular, biliary, renal, rejection, etc); they were graded according to Clavien grading. b- Early (less than 3months), and late (≥3months) mortalities, as well as mortality causes. c- Graft and patient survival outcomes.

The statistical analysis was done by SPSS 21 software (SSPS Inc, Chicago, IL, USA). Nominal variables were expressed in frequencies and percentages and analyzed using Fisher exact or Chi-square tests. Continuous variables were expressed as medians (ranges) or means±SDs and were compared using the t- or Mann-Whitney U tests. Univariate and then multivariate analyses were performed to detect predictors of early and/or late morbidities as well as predictors of patients' survival. The Kaplan–Meier method was applied for analysis of the survival of recipients and was compared using log-rank tests. In all tests, a P-value of <0.05 was significant.

## 3 Results

### 3.1 Pediatric recipients' characteristics

Regarding pre-operative recipients' and their donors' details; they were classified as 40(59.7%) males and 27(40.3%) females. Their median age and weight reached 2.8 (range; 0.7–17) years and 13 (range; 6.4–74) kg respectively. Their donors were categorized into 29(43.3%) males and 38(56.7%) females, where, their median age and body mass index were 30 (range; 19.5–43) years and 26(range; 18–35) respectively. Table 1.

**Table 1:** The pre-operative recipients' and their donors' details.

Category	No (%) 67(100%) Or Median(range)
Donor age(years) (Median(range))	30(19.5–43)
Donor gender	
males	29(43.3%)
females	38(56.7%)
BMI of the donor (Median(range))	26(18–35)
Donor to recipient relation	
1st degree	52(77.6%)
2nd degree	3(4.5%)
3rd degree	2(3%)
4th degree	2(3%)
Unrelated	8(11.9%)
Recipient age(years) (Median(range))	2.8(0.7–17)
Recipient age >1year	5(7.5%)
Recipient weight(Median(range))	13(6.4–74)
Recipient weight>10 Kg	19(28.4%)
Recipient gender	
males	40(59.7%)
females	27(40.3%)
Metabolic disease as indication for LT	25(37.3%)
BA as indication for LT	23(34.3)
MELD score(≤12years) (Median(range))	16(11–26)
PELD score(>12years) (Median(range))	13.5(2–34)
PELD or MELD scores(Median(range)	14(2–34)
CTP score	
A	18(26.9%)
B	22(32.8%)
C	27(40.3%)
Pre LT PHN	40(59.7%)
Upper endoscopy result	
Not done	18(26.9%)
Free	9(13.4%)
PHG	10(14.9%)
OV grade I	15(22.4%)
OV grade II	8(11.9%)
OV grade III	4(6%)
OV grade IV	3(4.5%)
Bl. Group	
Compatible	19(28.4%)
Identical	48(71.6%)
No of LT/year(Median(range)	4(0–13)

BMI: Body mass index, LT: Liver transplantation, BA: Biliary atresia, MELD: Model for end-stage liver disease, PELD: Pediatric end-stage liver disease, CTP: Child-Turcotte-Pugh, PHN: Portal hypertension, PHG: Portal hypertensive gastropathy, OV: Esophageal varices.

The 1st-degree donor to recipient relation was the most frequent (77.6%). The recipients' median MELD and PELD scores were 16(range; 11–26) and 13.5(range; 2–34) respectively, moreover, their most frequent Child-Turcotte-Pugh (CTP) score was C 27(40.3%). Pre LT Portal hypertension (PHN) affected 40(59.7%) of them, in whom; oesophagal varices (OV) grade I was the most frequent endoscopic finding 15(22.4%). The identical donor to recipient blood group matching was more frequent 48(71.6%). Lastly, the median NO of LT/year was 4(range; 0–13) cases; Table 1. The most frequent primary liver disease that has led to LT was biliary atresia (34.3%), followed by Byler's disease (17.9%); Table 2.

**Table 2:** The primary pediatric liver disease.

Category	No (%) 67(100%)
BA	23(34.3%)
Byler's disease	12(17.9%)
Cryptogenic liver cirrhosis	6(9%)
Crigler Najjar syndrome	5(7.5%)
AIH	3(4.5%)
BCS	3(4.5%)
Wilson's disease	2(3%)
Secondary biliary cirrhosis from choledochal cyst	2(3%)
Congenital hepatic fibrosis	2(3%)
HCV	2(3%)
Tyrosinemia	2(3%)
Hepatoblastoma	2(3%)
cavernous haemangiomas	1(1.5%)
Bile Ducts Paucity	1(1.5%)
Primary Hyperoxaluria	1(1.5%)

BA: Biliary atresia, AIH: Autoimmune hepatitis, BCS: Budd Chiari syndrome, HCV: Hepatitis C virus.

As regards recipients' operative variables details; 56(83.6%), 6(9%), 4(6%), and 1(1.5%) of patients were given left lateral, left lobe + middle hepatic vein (LL + MHV), (right lobe (RL)-MHV), and mono-segment II liver grafts respectively. The single HV, PV, HA and biliary anastomoses were done in 64(95.5%), 67(100%), 61(91%) and 55(82.1%) patients respectively while <1 anastomosis of the HV, HA and bile duct were performed in 3(4.5%), 6(9%) and 12(18%) of them respectively. The duct to duct (D-D) and hepaticojejunostomy(HJ) biliary reconstructions were performed in 29.9% and 70.1% of our recipients respectively, furthermore, biliary stents were put in 88.1% of them. The median actual graft weight and GRWR were 300 (range; 110–900) gm and 2 (range; 0.7–4.3) respectively. However, actual GRWR≥3, GRWR<4 (LFSG), and GRWR<0.8(SFSG) were 15(22.4%), 2(3%), and 1(1.5%) of patients respectively. The median cold and warm ischemia times (CIT and WIT) were 45 (range; 10–105) mins and 35 (range; 25–80) mins respectively. The median intra-operative packed Red blood cells (RBCs) and plasma transfusions were 1.5(range; 0–5) units and 3(range; 0–12) units respectively. The median operative time and postoperative hospital stay were 9 (range; 5–14) hours and 23 (range; 1–135) days respectively.Table 3.

**Table 3:** The intra-, and post-operative details.

Category	No (%) 67(100%) Or Median(range)
Graft type	
Left lateral	56(83.6%)
LL + MHV	6(9%)
RL-MHV	4(6%)
Monosegment II	1(1.5%)
HV anastomosis NO	
1	64(95.5%)
2	2(3%)"
3	1(1.5%)
PV anastomosis NO	
1	67(100%)
HA anastomosis NO	
1	61(91%)
2	6(9%)
Biliary anastomosis type	
D-D	20(29.9%)
HJ	47(70.1%)
Biliary anastomosis NO	
1	55(82.1%)
2	12(17.9%)
Biliary stent	59(88.1%)
Actual graft weight(g) (Median(range))	300(110–900)
Actual GRWR (Median(range))	2(0.7–4.3)
Actual GRWR≥3	15(22.4%)
Actual GRWR>4(LFSG)	2(3%)
Actual GRWR<0.8(SFSG)	1(1.5%)
CIT (min) (Median(range))	45(10–105)
WIT (min) (Median(range))	35(25–80)
Intraoperative packed RBCs (units) (Median(range))	1.5(0–5)
Intraoperative packed RBCs ≥2units	20(29.9%)
Intraoperative plasma transfusion(units) (Median(range))	3(0–12)
Operative time (hours) (Median(range))	9(5–14)
Post operative hospital stay(days) (Median(range))	23(1–135)
Immunosuppression regimen	
Regimen including FK	67(100%)
interleukin-2 receptor blocker	20(29.9%)
Regimen including MMF	14(20.9%)
Regimen including Cyclosporine	4(6%)
Regimen including Sirolimus	4(6%)
Regimen including Everolimus	2(3%)

LL: Left lobe, MHV: Middle hepatic vein, RL: Right lobe, HV: Hepatic vein, NO: Number, PV: Portal vein, HA: Hepatic artery, D-D: Duct to duct, HJ: Hepaticojejunostomy, GRWR: Graft recipient weight ratio, LFSG: Large for size graft, SFSG: Small for size graft, CIT: Cold ischemia time, WIT: Warm ischemia time, RBCs: Red blood cells, FK: Tacrolimus, MMF: Mycophenolate mofetil.

### 3.2 Postoperative morbidities

Forty-one (61.2%) of our pediatric recipients had ≥1 early (less than 3months) and/or late (≥3months) morbidities. Regarding early morbidities; they affected 36(53.7%) of patients where early bacterial infections were the most frequent ones; they affected 21(31.3%) of patients (24 infections involved 21 patients). They were classified into chest infection (11(16.4%)), biliary infection (5(7.5%)), wound infection (7(10.5%)) and infected abdominal collection (1(1.5%)); moreover, they were categorized into Clavien grades II, III, and V in 9, 2 and 13 of them respectively. They were managed by antibiotics according to culture and sensitivity, by intervention radiology and/or surgically. The treatment was successful in 11 of those infections. Table 4.

**Table 4:** Early morbidities.

Category	Clavien grade II	Clavien grade IIIa or b	Clavien grade V	Treatment result (Success)	Treatment result (Failure)	Total No (%) 67(100%)
Early complications (<3months)						36(53.7%)
Bacterial infection						21(31.3%)
1-Chest infection	1	0	10	1	10	11(16.4%)
2-Biliary infection	2	0	3	2	3	5(7.5%)
4-Wound infection	6	1	0	7	0	7(10.5%)
5-Infected abdominal collection(perforated colon)	0	1	0	1	0	1(1.5%)
Pulmonary complications						13(19.4%)
1-Chest infection	1	0	10	1	10	11(16.4%)
2-Pulmonary embolism	0	0	1	0	1	1(1.5%)
3-Hemothorax	0	1	0	1	0	1(1.5%)
Acute rejection	9	0	1	9	1	10(14.9%)
Vascular						8(12%)
1-HA stenosis	0	0	1	0	1	1(1.5%)
2- HAT	0	0	1	0	1	1(1.5%)
3- PVT	1	0	1	1	1	2(3%)
4-HV stenosis	0	1	0	1	0	1(1.5%)
5-PVT + HVT	0	0	2	0	2	2(3%)
6-IVC stenosis	0	0	1	0	1	1(1.5%)
Biliary						7(10.5%)
1-Bile leak + biloma	0	1	1	1	1	2(3%)
2- Bile leak	0	1	2	1	2	3(4.5%)
3-Cholangitis	2	0	0	2	0	2(3%)
Wound complications						7(10.5%)
1-Wound infection + burst abdomen	0	1	0	1	0	1(1.5%)
2-Wound infection	6	0	0	6	0	6(9%)
Renal impairment	3	0	2	3	2	5(7.5%)
GIT complications						4(6%)
1-Haematemesis	0	2	0	2	0	2(3%)
2-Colonic perforation	0	1	0	1	0	1(1.5%)
3-Hepatic encephalopathy	0	0	1	0	1	1(1.5%)
Neurological complications	1	0	0	1	0	1(1.5%)
Recurrent BCS	0	0	1	0	1	1(1.5%)
Early graft failure	0	0	1	0	1	1(1.5%)

HA: Hepatic artery, HAT: Hepatic artery thrombosis, PVT: Portal vein thrombosis, HV: Hepatic vein, HVT: Hepatic vein thrombosis, IVC: Inferior vena cava, GIT: Gastrointestinal, BCS: Budd Chiari syndrome.

Early pulmonary complications affected 13(19.4%) of our recipients and were categorized into chest infection (11(16.4%)), pulmonary embolism (1(1.5%)) and hemothorax(1(1.5%)). As regards Clavien's grading; grades II, III and V affected 1, 1 and 11 of them respectively. They were managed as follows: Antibiotics for the chest infection, anticoagulants for pulmonary embolism and a chest tube for hemothorax with successful treatment in 2 of them only. Table 4.

Early acute rejection affected 10(14.9%) of our paediatrics. Clavien grades II and V involved 9 and 1 of them respectively. They were managed by pulse steroids where 4, 3, 1 and 2 of patients were given 1, 2, 3 and 4 boluses respectively with good outcomes in 9 of them. Table 4.

Eight (12%) of our patients had early vascular complications that were sorted into HA stenosis (1(1.5%)), HA thrombosis (HAT) (1(1.5%)), PV thrombosis (PVT) (2(3%)), HV stenosis (1(1.5%)), PVT + HV thrombosis (HVT) (2(3%)) (from LFSG) and IVC stenosis (1(1.5%)). Clavien grades II, III and V involved 1, 1 and 6 of them respectively. They were managed medically (anticoagulants or thrombolytic therapy), by angiography (dilatation and/or stenting and/or thrombolytic therapy) and/or by surgery (thrombectomy and/or re-anastomosis) with good outcome in 2 of them only. Table 4.

The incidence of early biliary complications was 7(10.5%) in the form of biliary leak + biloma, biliary leak and cholangitis in 2(3%), 3(4.5%) and 2(3%) of patients respectively. Regarding Clavien grading; grades II, III and V involved 2, 2 and 3 of them respectively. The cases with biliary leak ± biloma were managed conservatively, by percutaneous drainage, endoscopic retrograde cholangeopancreatography (ERCP) and/or surgery (Open drainage, and/or external biliary diversion) under antibiotic coverage; however cholangitis cases were managed by antibiotics with a good result in 4 of the 7 patients. Table 4.

Early wound complications affected 7(10.5%) of patients in the form of wound infection and wound infection + burst abdomen in 6(9%) and 1(1.5%) of patients respectively. They were managed medically by antibiotics for the infection or surgically for burst abdomen. They were in the category of II and III regarding Clavien grades with excellent results in all of them. Table 4.

Five (7.5%) of our patients had early renal impairment where Clavien grade II and V affected 3 and 2 of them respectively; they were managed by renal supportive treatment with good outcomes in 3 of them. Table 4.

Early gastrointestinal (GIT) complications in the form of haematemesis, colonic perforation and hepatic encephalopathy affected 2(3%), 1(1.5%) and 1(1.5%) of our transplanted children respectively. They were managed by endoscopy, surgically and by medical treatment respectively with a successful outcome in 3 of them. Table 4.

Finally; early neurological complications affected 1(1.5%) of our patients who underwent neurological support with a good outcome, lastly, we had a case with recurrent Budd Chiari syndrome (BCS) (IVC stenosis) and another case with early graft failure, they were managed by angiographic dilatation, and liver support respectively but unfortunately; both cases died.Table 4.

As regards late morbidities; they affected 12(17.9%) of our patients. Late bacterial infections involved 4(6%) of our smart recipients; those infections were classified into chest, and biliary infections that affected 3(4.5%) and 1(1.5%) of patients respectively, furthermore, they were sorted regarding Clavien grading into grades II, III and V in 1, 1 and 2 of them respectively. They were managed by antibiotics; moreover, the cholangitis case was managed surgically. The outcome was successful in 2 of the 4 cases. Table 5.

**Table 5:** Late morbidities.

Category	Clavien grade II	Clavien grade IIIa or b	Clavien grade V	Treatment result (Success)	Treatment result (Failure)	Total No (%) 67(100%)
Late complications (≥3months)						12(17.9%)
Bacterial infection						4(6%)
1-Chest infection	1	0	2	1	2	3(4.5%)
2-Biliary	0	1	0	1	0	1(1.5%)
Chronic rejection	0	0	4	0	4	4(6%)
Pulmonary complications						4(6%)
1-Chest infection	1	0	2	1	2	3(4.5%)
2-Pleural effusion	0	1	0	1	0	1(1.5%)
Biliary						4(6%)
1-HJ stricture + recurrent cholangitis	0	1	0	1	0	1(1.5%)
2-HJ stricture	0	1	0	1	0	1(1.5%)
3- D-D stricture	0	2	0	2	0	2(3%)
Vascular						3 (4.5%)
1- HAT	1	0	0	1	0	1(1.5%)
2-HV stenosis	0	1	1	1	1	2(3%)
Acute rejection	2	0	0	2	0	2(3%)
Renal impairment	1	0	1	1	1	2(3%)
Recurrent BCS	0	1	0	1	0	1(1.5%)

HJ: Hepaticojejunostomy, D-D: Duct to duct, HAT: Hepatic artery thrombosis, HV: Hepatic vein, BCS: Budd Chiari syndrome.

The incidence of chronic rejection was 4(6%) that occurred in the 11th, 12th, 16th and 18th post-transplant months. It was diagnosed histologically according to updated Banff criteria [31]**.** Patients were given MMF beside FK for its management, furthermore, when FK toxicity occurred they were shifted to Sirolimus or Everolimus, however, the 4 patients, unfortunately, died (Clavien grade V).Table 5.

In our series, the late pulmonary complications involved 4(6%) of recipients, they were divided into chest infections (3(4.5%) and pleural effusions (1(1.5%), moreover, they were categorized into Clavien grades II, III and V in 1, 1 and 2 of them respectively; they were managed by antibiotics for infection and chest tube for effusion with 2 mortalities; one from sepsis and the other from acute respiratory distress syndrome (ARDS). Table 5.

Four (6%) of our pediatric patients had late biliary complications in the form of HJ stricture + cholangitis, HJ stricture and D-D stricture that involved 1(1.5%), 1(1.5%) and 2(3%) of them respectively; they were all Clavien grade III as they were managed by ERCP, percutaneous transhepatic drainage (PTD) and/or surgical reconstruction under antibiotic coverage with final improvement in all of them. Table 5.

As regards late vascular complications; they were 3 cases (4.5%) and were classified into HAT and HV stenosis in 1(1.5%) and 2(3%) of them respectively. Clavien grades II, III and V involved 1, 1 and 1 of them; they were managed by anticoagulants and fibrinolytic for HAT as well as by angiographic dilatation and stenting for HV stenosis with successful results in 2 of the 3 cases. Table 5.

Lastly, late acute rejection, renal impairment and recurrent BCS affected 2(3%), 2(3%) and 1(1.5%) of patients respectively; they were managed by pulse steroids for rejection, renal supportive treatment for renal impairment and angiographic dilatation and stenting for BCS with a successful outcome in 2, 1 and 1 of them respectively. Table 5.

### 3.3 Predictors of early and/or late morbidity

On univariate analysis, CTP class C, higher PELD/MELD scores, biliary stents, more intra-operative packed RBCs transfusion units and longer duration of operation were predictors of early and/or late morbidities, however, on multivariate analysis, there was no independent predictor of those morbidities.Table 6.

**Table 6:** Predictors of early and/or late morbidities.

Category	Early and/or late morbidity No (%) 41 (100%) or (Mean ± SD)	No morbidity No (%) 26(100%) or (Mean ± SD)	P-value Univariate analysis	P-value Multivariate analysis
Recipient age(year)	5.1 ± 5.2	6.2 ± 5.6	0.4	
Recipient age <1year	4(9.8%)	1(3.8%)	0.4	
Recipient weight(kg)	18.6 ± 15.6	22.2 ± 18.7	0.4	
Recipient weight <10 kg	13(31.7%)	6(23.1%)	0.4	
CTP score			0.029	0.9
A	7 (17.1%)	11(42.3%)		
B	13(31.7%)	9(34.6%)		
C	21(51.2%)	6(23.1%)		
PELD or MELD scores	16.3 ± 7.3	11.4 ± 5.5	0.006	0.2
Pre LT PHN	26(63.4%)	14(53.8%)	0.3	
Actual graft weight(g)	311.9 ± 137.8	378.1 ± 197.8	0.2	
Actual GRWR	2.2 ± 0.9	2.1 ± 0.8	0.7	
Actual GRWR≥3	10(24.4%)	5(19.2%)	0.4	
Biliary stent	39(95.1%)	20(76.9%)	0.033	0.1
CIT (min)	54.4 ± 27	50 ± 22.6	0.5	
WIT (min)	38.1 ± 10	40.2 ± 14.1	0.5	
Intraoperative packed RBCs transfusion (units)	1.7 ± 1	1.1 ± 1.2	0.003	0.1
Intraoperative plasma transfusion(units)	3.6 ± 3	2.4 ± 2.3	0.1	
Operative time (hours)	9.4 ± 2	8 ± 1.9	0.006	0.1

CTP: Child-Turcotte-Pugh, PELD: Pediatric end-stage liver disease, MELD: Model for end-stage liver disease, LT: Liver transplantation, PHN: Portal hypertension, GRWR: Graft recipient weight ratio, CIT: Cold ischemia time, WIT: Warm ischemia time, RBCs: Red blood cells.

### 3.4 Survival outcomes of pediatric patients

**Fig. 6:**
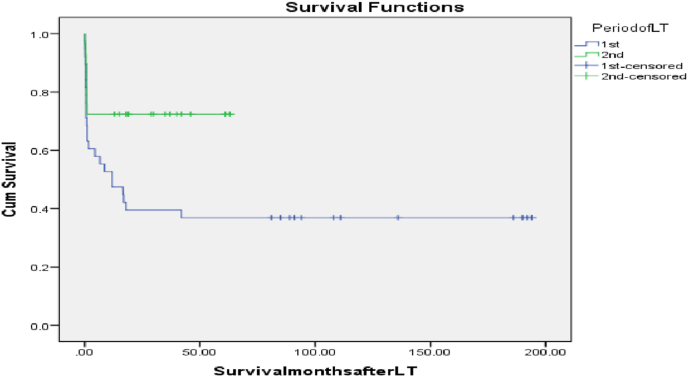
Kaplan-Meier patient survival curve: Period of LT and survival; 1st (2003–2013), 2nd (2014–2018) ((Log rank = 0.013).

In our work; the 6months, 1-year, 3-year, 5-year, 10-year and 16-year graft and patient survivals were 42(62.7%), 39(58.2%), 36(53.7%), 35(52.2%), 35(52.2%) and 35(52.2%), and 43(64.2%), 41(61.2%), 36(53.7%), 35(52.2%), 35(52.2%) and 35(52.2%) respectively. The mortality in the 1st LT period ((from 2003 to 2013); 38 patients) reached 63.2%, however it was significantly less (27.6%; p = 0.004) in the 2nd LT period (from 2014 to 2018); 29 patients). The early (less than 3months) mortality reached 34.3% mostly due to sepsis, renal impairment and LFSGs; however, the late (≥3months) mortality was 13.4% mostly from chronic rejection and late sepsis. Table 7; Fig. 6.

**Table 7:** Recipients' survival outcome.

Category	No (%) 67(100%)
Graft survival	
6 months survival	42(62.7%)
1-year survival	39(58.2%)
3-year survival	36(53.7%)
5-year survival	35(52.2%)
10-year survival	35(52.2%)
16-year survival	35(52.2%)
Patient survival	
6 months survival	43(64.2%)
1-year survival	41(61.2%)
3-year survival	36(53.7%)
5-year survival	35(52.2%)
10-year survival	35(52.2%)
16-year survival	35(52.2%)
Survival per months Median(Range)	18(0.03–194)
a1st-period mortality	24/38(63.2%)
2nd-period mortality	8/29(27.6%)
Early mortality(3months)	23(34.3%)
Main causes:	
Sepsis	12(17.9%)
Renal impairment	2(3%)
LFSG	2(3%)
Hepatic encephalopathy	1(1.5%)
Acute rejection	1(1.5%)
Early graft failure	1(1.5%)
HAT	1(1.5%)
PVT	1(1.5%)
IVC stenosis	1(1.5%)
Pulmonary embolism	1(1.5%)
Late mortality	9(13.4%)
Main causes:	
Chronic rejection	4(6%)
Sepsis	2(3%)
ARDS	1(1.5%)
HV stenosis	1(1.5%)
Renal impairment	1(1.5%)

a Difference is significant, 1st-period mortality: From (2003–2013), 2nd-period mortality (from 2014 to 2018), LFSG: Large for size graft, HAT: Hepatic artery thrombosis, PVT: Portal vein thrombosis, IVC: Inferior vena cava, ARDS: Acute respiratory distress syndrome, HV: Hepatic vein.

### 3.5 Pre- and intra-operative parameters as predictors of patient survival outcome

**Fig. 7:**
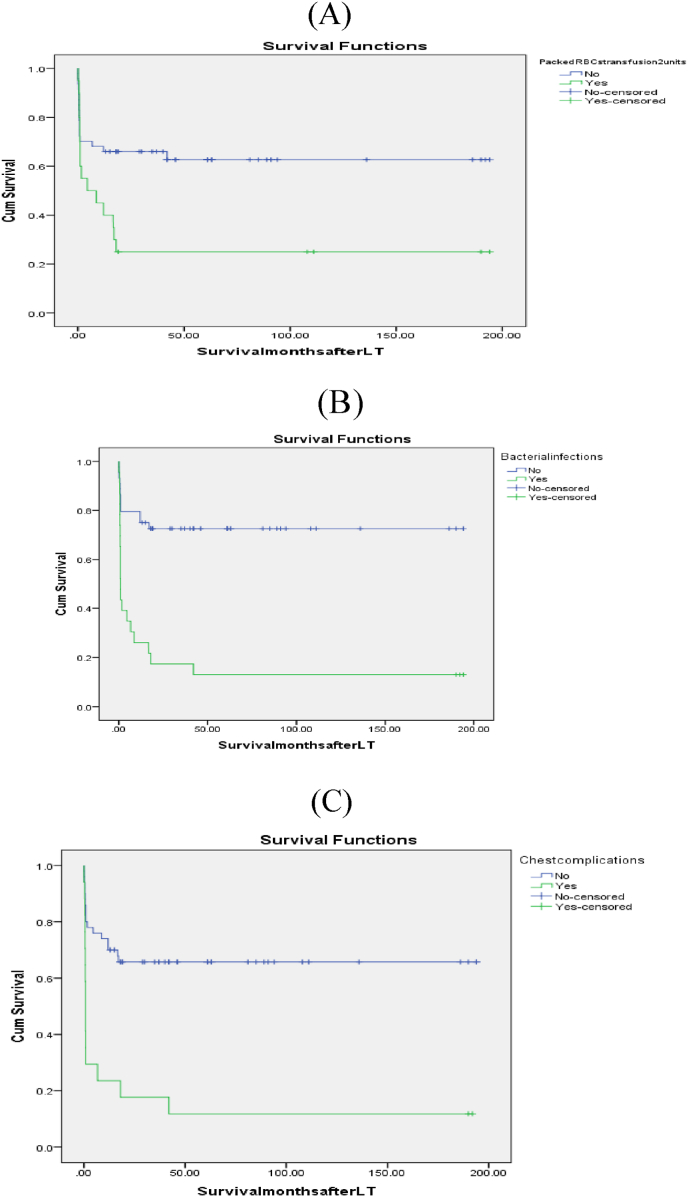
Kaplan-Meier patient survival curves. A: Packed RBCs transfusion ≤2units and survival (Log rank = 0.014).B: Bacterial infection and survival (Log rank = 0.000).C: Chest complications and survival (Log rank = 0.000).

On univariate analysis, CTP class C, Pre LT PHN, biliary stents, more intraoperative Packed RBCs transfusion units and longer duration of operation were predictors of poor patient survival, however, on multivariate analysis, GRWR≥3 had a trend towards independent correlation with patient mortality; moreover, more units of transfused Packed RBCs had an independent association with patient mortality. Table 8; Fig. 7.

**Table 8:** Pre- and intra-operative variables as predictors of patient survival outcome.

Category	Patient survival No (%) 35 (100%) or (Mean ± SD)	Patient mortality No (%) 32(100%) or (Mean ± SD)	P-value Univariate analysis	P-value Multivariate analysis
Recipient age(year)	6.4 ± 5.7	4.5 ± 4.9	0.06	0.6
Recipient age <1year	1(2.9%)	4(12.5%)	0.2	
Recipient weight(kg)	22.5 ± 18.3	17.1 ± 14.9	0.2	
Recipient weight <10 kg	9(25.7%)	10(31.3%)	0.4	
Metabolic cause as a primary disease	15(42.9%)	10(31.3%)	0.2	
BA as a primary disease	10(28.6%)	13(40.6%)	0.2	
CTP score				
A	12 (34.3%)	6(18.8%)	0.01	0.6
B	15(42.9%)	7(21.9%)		
C	8(22.9%)	19(59.4%)		
MELD score (>12years)	14.9 ± 3.1	19 ± 5.7	0.3	
PELD score (<12years)	12.4 ± 7.5	15.8 ± 7.1	0.1	
PELD/MELD scores	13.05 ± 6.7	16.2 ± 6.9	0.066	1
Pre LT PHN	17(48.6%)	23(71.9%)	0.045	0.7
Actual graft weight(g)	371 ± 202.5	309 ± 103.3	0.08	0.1
Actual GRWR	2.02 ± 0.7	2.3 ± 0.9	0.2	
Actual GRWR≥3	5(14.3%)	10(31.3%)	0.085	0.08
Biliary stent	27(77.1%)	32(100%)	0.004	1
CIT (min)	50.8 ± 21.7	54.8 ± 28.9	0.5	
WIT (min)	40.3 ± 13.4	37.3 ± 9.4	0.3	
Intraoperative packed RBCs transfusion (units)	1.2 ± 1.2	1.7 ± 0.8	0.001	0.04
Intraoperative plasma transfusion(units)	2.8 ± 2.7	3.5 ± 2.8	0.3	
Operative time (hours)	8.3 ± 1.8	9.4 ± 2	0.02	0.7

BA: Biliary atresia, CTP: Child-Turcotte-Pugh, MELD: Model for end-stage liver disease, PELD: Pediatric end-stage liver disease, LT: Liver transplantation, PHN: Portal hypertension, GRWR: Graft recipient weight ratio, CIT: Cold ischemia time, WIT: Warm ischemia time, RBCs: red blood cells.

### 3.6 Early and/or late morbidities as predictors of patient survival outcomes

On univariate analysis, the overall early and/or late morbidities, bacterial infections, pulmonary complications, acute rejection, chronic rejection, vascular complications, and renal impairment were significant risks of patient mortality, on the other hand, on multivariate analysis, bacterial infections and pulmonary complications were independent predictors of poor patient outcome. Table 9; Fig. 7.

**Table 9:** Early and late morbidities as predictors of patient survival outcome.

Category	Patient survival No (%) 35 (100%)	Patient mortality No (%) 32(100%)	P-value Univariate analysis	P-value Multivariate analysis
Early and/or late morbidities	9(25.7%)	32(100%)	0.000	
cBacterial infection	3(8.6%)	20(62.5%)	0.000	S
cPulmonary complications	2 (5.7%)	15(46.9%)	0.000	S
bAcute rejection	3(8.6%)	9(28.1%)	0.038	NS
bChronic rejection	0	4(12.5%)	0.047	NS
aBiliary complications	4(11.4%)	6(18.8%)	0.3	
aVascular complications	2(5.7%)	9(28.1%)	0.015	NS
aRenal impairment	1(2.9%)	6(8.8%)	0.040	NS
aBCS	1(2.9%)	1(3.1%)	0.7	

a Means early and/or late, BCS: Budd Chiari syndrome.

b Acute and chronic rejections were correlated so multivariate analysis was done for them separately.

c Bacterial and chest infections were correlated so multivariate analysis was done for them separately, S: Significant, NS: Non-significant.

## 4 Discussion

Despite being a challenging procedure; pediatric LDLT is a life-saving option for paediatrics with ESLD, and other catastrophic liver conditions like tumours especially in countries like Egypt that don't have a deceased donor liver transplantation (DDLT); however, the complications after it are still a big problem with devastating effects despite the recent improvement in such a field of pediatric LDLT [32].

Our early and/or late morbidities that affected 41(61.2%) of our patients lie within the literature range of post pediatric LDLT morbidities (15%–89.9%) [2,8,[33], [34], [35]].

Regarding individual complications; our biliary and vascular complications reached 14.9% and 16.4% respectively; similarly, the previous literature ranges of post pediatric LDLT biliary and vascular complications were 3.4%–42% [23,33,34,36,37] and 4.5%–43% [23,[33], [34], [35],^38^,^39^] respectively.

The post pediatric LDLT acute rejection ranged from 16% to 67% in different literature studies [23,[39], [40], [41], [42]]; however, the literature range of chronic rejection was 2.5%–8% [39,40,42,43]. Our rates of acute and chronic rejections were within those previous literature ranges as they reached 17.9% and 6% respectively.

Renal dysfunction after pediatric LT ranged from 10.4% to 31.6% in Kehar et al., 2019 [32] and Campbell et al., 2006 [44] studies. Also, it was 10.4% in our work. On the other hand, post pediatric LT pulmonary complications were 25.4%, 49%, and 86% in ours, Alam, et al., 2017 [45] And Ruchonnet-Metrailler et al., 2018 [46] studies respectively.

Despite advanced infection control policies and antibacterial prophylaxis; bacterial infections that cause remarkable morbidities and mortalities in the early and late periods after pediatric LT are still common due to poor patient general condition, being ultra major operation, using immunosuppressants, etc [16,39]. They ranged from 39.1% to 67% in the previous literature [39,42,and47]], however, they were less in our series (34.3%); and this is due to our improved infection control policies, especially in our later cases.

Higher PELD/MELD scores were associated with morbidities in our work, in the same line; they were significant or independent predictors of morbidities in Kitajima et al., 2017 [2], Raices, et al., 2019 [8], and Chung et al., 2020 [48] studies, moreover, they were independent predictors of early re-laparotomy due to morbidities in Okada et al., 2019 [49] study.

Longer operative time was a significant predictor of morbidity in our present work, also, it was associated with re-laparotomies due to morbidities in Yoeli et al., 2018 [6] and Okada et al., 2019 [49] studies.

Increased amounts of intra-operative packed RBCs transfusion was a predictor of morbidity in the present series, in similar, it was correlated with relaparotomy from morbidities in Yoeli et al., 2018 [6] work.

Our 5-, 10-, and 16-year post-transplant patient survival were 52.2%, 52.2%, and 52.2% respectively, however, the literature ranges of post pediatric LDLT 5-, 10-, and 20- year patient survivals were 69%–97% [23,43,[50], [51], [52]], 77.2%–94% [32,42,51,53,54], and 79.6%–84.2% [51,55] respectively. On the other hand, our patient mortality reached 47.8%; however, it ranged in the pediatric LDLT literature between 4.2% and 13% [2,32,34,56,57]. Our lower survival and higher mortalities in comparison to the literature come from several reasons: 1- most mortalities occurred within the 1st 3months post LT(23/32; 72%) due to sepsis that decreased in later cases after improving infection control policies.2- We are a mixed adult/pediatric LT centre with few pediatric LT cases/year(median 4; range(0–13) cases; low volume pediatric LT centre)3-The mortality was higher in the earlier periods of LT due to less experience but improved in later periods.

Higher PELD/MELD scores had a trend towards significant correlation with poor patient survival in our work, also, a higher PELD score was an independent predictor of poor patient survival in Pan et al., 2020 [22], Lu, et al., 2020 [25] and Kehar et al., 2019 [32] studies**,** moreover, it was a significant predictor of patient loss in Oh et al., 2010 [42]; study, in contrast, it did not affect survival in Kitajima et al., 2017 [2], Raices, et al., 2019 [8], Chung, et al., 2020 [48] or Shehata et al., 2012 [58] studies.

Post LDLT bleeding that comes from technical issues, collaterals, and bleeding tendency has a negative insult on survival outcomes [7]. Similarly, increased intraoperative blood loss was associated with patients' mortalities in Pan et al., 2020 [22] and Lu et al., 2020 [25] studies. Furthermore, More RBC units' transfusion was correlated with patient mortality in our and Boillot et al., 2021 [24] studies. Conversely; increased intra-operative blood loss was not associated with patient survival in Shehata et al., 2012 [58] study.

Longer operative time was a significant predictor of patient mortality in our study, also, it was an independent predictor of poor patient survival in Pan et al., 2020 [22] study, however; it was not associated with survival in Boillot et al., 2021 [24] or Shehata et al., 2012 [58] studies.

Due to accumulating experience, surgical techniques advancement, and pre-and post-transplant care improvement; the later periods of LT have been better than the earlier ones regarding patient survival, in similar, later periods of LT were significant predictors of better survival in ours, Pan, et al., 2020 [22], Pu, et al., 2020 [23], Boillot, et al., 2021 [24] and Venick et al., 2018 [59] studies, but they did not affect survival in Raices et al., 2019 [8] or Shehata et al., 2012 [58] studies.

The overall post-transplant complications were associated with patient mortality in the recent study, also, complications were significant predictors of mortality in Ho et al., 2004 [7] study, and early relaparotomy from early morbidities was significantly associated with poor patient survival in Okada et al., 2019 [49] study.

LFSG affects post LT outcomes by reducing oxygen and blood supplies of the liver graft, increasing vascular complications rate, inducing allograft dysfunction and/or loss and/or necrosis, inducing renal dysfunctions, as well as the occurrence of ACS and large for size syndrome [8,23]**.** In a similar line in our work; The 2 cases with LFSGs died from their sequels, and GRWR≥3 had a trend towards independent correlation with patient mortality, also; LFSG was an independent predictor of poor patient survival in Lu et al., 2020 [25] study**.** However, it did not affect survival in Kitajima et al., 2017 [2], Goldaracena, et al., 2020 [33], Ersoy, et al., 2017 [56], Shehata, et al., 2012 [58] or Akdur et al., 2015 [60] studies.

Despite being a cause of morbidity and mortality after LT; biliary complications did not affect mortality in ours, Liao, et al., 2019 [18] or Sanada et al., 2019 [37] studies.

Post pediatric LDLT vascular complications are common causes of morbidity and mortality [39]. Also, they were predictors of patients' mortality in ours, Steinbrück, et al., 2011 [61] and Sieders et al., 2000 [62] studies. But they did not affect survival in Shehata et al., 2012 [58] study.

Post LT acute rejection is a known cause of graft dysfunction [63]. It was correlated with patient loss in our series; also, it was a major cause of death in Kitajima et al., 2018 [34] study. However, it did not affect graft or patient survival in Yilmaz et al., 2006 [40] or Shehata et al., 2012 [58] studies.

Despite advanced immunosuppression after LT; chronic rejection remains a major reason for graft and/or patient loss [42,43,59,64]. Also, in our work; it was the major cause of late mortality and a significant predictor of patient loss, similarly, it was an independent predictor of patient loss in Oh et al., 2010 [42] study, In contrast, it did not affect graft or patient survival in Yilmaz et al., 2006 [40] study.

We found a significant correlation between post LT renal impairment and patient mortality, similarly; Post LT hemodialysis was an independent predictor of poor patient survival in Boillot et al., 2021 [24] study. Regarding pulmonary complications; they were associated with patient mortality in Alam et al., 2017 [45] study. Also, they were independently associated with mortality in our work.

Bacterial infection was associated with patient mortality in Pouladfar et al., 2019 [47] and Shepherd et al., 2008 [65] studies. Also, it was an independent predictor of patient loss in our study and in a similar line; sepsis was the major cause of early mortality, and the 2nd most common cause of late mortality in our study, similarly; it was the major cause of mortality in Kitajima et al., 2017 [2], Kehar, et al., 2019 [32], Kitajima, et al., 2018 [34], Mohan, et al., 2017 [43] and Tanaka et al., 2010 [66] pediatric LDLT studies.

Lastly, to our knowledge; this is one of the unique pediatric LDLT studies mentioning the independent association between both pulmonary complications and bacterial infection and patient mortality, and this is due to sepsis which has led to those catastrophic mortalities. In conclusion; more packed RBCs transfusion units intra-operatively, and post LT bacterial infection, sepsis, chronic rejection, as well as pulmonary complications had a negative insult on our patients' outcomes, so proper management of them is mandatory for improving outcomes after pediatric LDLT.

## Ethical approval

The approval by National liver institute (IRB), Menoufia University that was done retrospectively.

## Sources of funding

No source of funding for this research.

## Author contributions

Emad Hamdy Gad: Surgical procedures, study design, data collection, writing, analysis and publication.

Ahmed Nabil Sallam: Surgical procedures, data collection, and analysis.

Hosam Soliman: Surgical procedures, and analysis.

Tarek Ibrahim: Surgical procedures, and analysis.

Tahany Abdel Hameed Salem: Study design, data collection, and writing.

Mohammed Abdel-Hafez Ali: Study design, data collection, and writing.

Mohammed abdelsamee: Data collection and writing.Islam Ayoub: Study design, surgical procedures, data collection and analysis.

## Guarantor

All the authors of this paper accept full responsibility for the work and/or the conduct of the study, had access to the data, and controlled the decision to publish.

## Provenance and peer review

Not commissioned, externally peer-reviewed.

## Declaration of competing interest

No conflict of interest to declare.
